# Academic Emotions, Emotion Regulation, Academic Motivation, and Approaches to Learning: A Person-Centered Approach

**DOI:** 10.3390/bs15070900

**Published:** 2025-07-03

**Authors:** Christos Rentzios, Evangelia Karagiannopoulou, Georgios Ntritsos

**Affiliations:** 1 Department of Psychology, Neapolis University of Cyprus, Paphos 8042, Cyprus; 2 Department of Psychology, University of Ioannina, 45110 Ioannina, Greece; ekaragia@uoi.gr; 3 Department of Clinical Educational and Healht Psychology, University College London, London WC1E 6BT, UK; 4 Department of Economics, School of Econonomics and Management Sciences, University of Ioannina, 45110 Ioannina, Greece; gntritsos@uoi.gr

**Keywords:** academic emotions, emotion regulation, academic motivation, approaches to learning

## Abstract

Contemporary educational literature suggests that academic emotions and emotion regulation should be explored in tandem, while academic motivation has been discussed both as a self-regulation metacognitive construct and as a construct inherently tied to motivation. The present study uses a person-centered approach to explore profiles of university students based on academic emotions, emotion regulation, academic self-regulation, and approaches to learning. In addition, the impact of students’ profiles on academic performance (GPA) is investigated. The sample consists of 509 university students studying at a Greek university social science department. Cluster techniques and multivariate analysis of variance are used to identify the profiles and test for differences among them. Students were grouped in clusters that revealed both consistent and dissonant patterns of scores on the relevant variables. Analysis reveals three distinct profiles: (a) the “Anxious, effectively-engaged, and organized learners”, (b) the “Deep, Happy, and intrinsically motivated learners” and (c) the “Disengaged, Bored, and Suppressing Learners”. These profiles open new insights into educational literature, revealing links among learning, emotional, and motivational factors. Practical implications and directions for future research are discussed.

## 1. Introduction

Over the past decade, there has been a significant increase in the number of studies that have examined the cognitive and emotional factors that contribute to learning in higher education (Vermunt & Donche, 2017). Consistent with the perspective put forward by Pekrun (2024b), we maintain that integrating constructs originating from different theoretical backgrounds is not only feasible, but essential for adequately addressing the multifaceted nature of learning. Contemporary studies in higher education emphasize a number of emotional factors that may facilitate or hinder student learning (Karagiannopoulou et al., 2020; Rentzios & Karagiannopoulou, 2021).

Motivation and emotions are crucial in learning and performance (Kim & Pekrun, 2014). While some scholars (Hannula, 2006) view emotions as a component of motivation processes, others (Op’t Eynde & Turner, 2006) view motivation as a component of emotional processes. Consequently, motivation and emotion are regarded as two facets of the same process (Kim & Pekrun, 2014). Thus, an integrative perspective on motivation and emotions is required to facilitate learning and performance (Kim & Hodges, 2012; Miele et al., 2024).

Moreover, given that both positive and negative emotions that students encounter in demanding settings, such as universities, may promote and/or hinder performance, it is reasonable for university students to attempt to regulate them (Harley et al., 2019). Literature suggests that academic emotions and emotion regulation strategies should be examined in tandem in order to elucidate the complexity and nuances of emotion regulation (ER) in achievement contexts (Harley et al., 2019). In particular, Harley et al. (2019) propose that various emotion regulation strategies can be implemented and integrated to manage emotions effectively in learning and educational contexts (Frenzel et al., 2024; Harley et al., 2019).

The present study employs a person-centered approach to acquire a deeper comprehension of student profiles, especially considering recent research that highlights an increase in dissonant profiles in educational environments (Parpala et al., 2022). This method provides a chance to explore the distinct characteristics of various student groups and their impact on both learning behavior and their academic performance. The simultaneous presence of these characteristics—namely, academic emotions, emotion regulation strategies, types of academic motivation, and approaches to learning—has not been previously examined using a profile-based approach, leading us to investigate this connection further. Most studies in the educational field examine students’ profiles focusing mainly on emotional (Robinson et al., 2017) or cognitive factors (Lonka et al., 2021; Tuononen et al., 2023); here, we propose a combination of both, adding the motivation facet. A person-centered approach has the potential to enhance our understanding of the complexities of intersecting components, therefore providing new insights into the interaction regarding emotional factors and learning (Karagiannopoulou et al., 2020; Milienos et al., 2021). Koenka (2020) suggests that using new data-analysis tools (e.g., person-centered approach) facilitates the detection of significant processes in learning. Moreover, in order to justify and enhance the extracted profiles, we consider the grade point average (GPA) as an outcome of these profiles to determine whether there are any significant differences in GPA across profiles.

The current study attempts to explore the joint co-occurrence of these crucial components of the learning process. That is, we explore emotion regulation strategies (reappraisal and suppression), academic emotions (enjoyment, anxiety and boredom), academic motivation-regulation (external, introjection, identification and intrinsic), and approaches to learning (deep, surface and organized).

### 1.1. Emotion Regulation

ER refers to the explicit or implicit attempts individuals engage to affect the feelings they experience, the timing of these emotions, and their expression and perception of them (Gross, 2015). A considerable amount of research has concentrated on cognitive reappraisal and suppression. Reappraisal comprises recontextualizing a situation to modify one’s emotional reaction, whereas suppression involves restraining the external manifestation of emotion (McRae & Gross, 2020). Reappraisal is frequently seen as adaptive, whereas suppression is considered maladaptive. Recent evidence, however, suggests that the context in which the emotion arises is critical (Rottweiler et al., 2018). In an educational setting, for example, suppression has been associated with beneficial outcomes in learning environments (Webster & Hadwin, 2015). Moreover, the consistent use of reappraisal has been demonstrated to correlate with increased positive emotions, reduced negative emotions during learning, increased motivation associated with learning, and improved self-regulation of learning, hence facilitating deeper knowledge acquisition and academic achievement (Losenno et al., 2020; Spann et al., 2019).

Furthermore, students seldom deploy reappraisal, reflecting Suri et al.’s (2015) conclusion that this approach is implemented less frequently than anticipated, despite its adaptiveness. Indeed, reappraisal may be highly demanding, particularly during situations of extreme stress. Consequently, it may not be beneficial in performance-focused contexts such as tests, which inherently place significant cognitive demands on students (Frenzel et al., 2024). While reappraisal is predominantly seen as a cognitive regulation skill, its increasing use across development may be supported by the maturation of social-cognitive processes, particularly during adolescence (McRae et al., 2012). The capacity for ER is linked to the activation of specific prefrontal brain regions that are crucial to cognitive control and executive functioning, which emerge later in the maturation process (Martin & Ochsner, 2016). On the other hand, suppressing emotions denotes a highly regulated kind of ER in which individuals deny and downplay the intensity and significance of their feelings regarding oneself (Kim & Pekrun, 2014; Roth et al., 2019). When experiencing suppression, individuals also feel forced to conceal their negative emotions towards others, thus inhibiting emotional expression (Roth et al., 2019). In general, reappraisal is more commonly utilized when emotional intensity is moderate, whereas suppression is employed when intensity is high (McRae & Gross, 2020).

According to Self-Determination Theory (SDT), the way individuals regulate their emotions is related to different types of motivation: e.g., autonomous motivation or controlled motivation (Ryan & Deci, 2017).

### 1.2. Academic Motivation

SDT is a macro-motivational theoretical framework that focuses on the traditional distinction between intrinsic and extrinsic motivation, and it has been extensively utilized in educational research (Ryan & Deci, 2020). SDT posits that the form of motivation among university students influences the extent of energy they may apply to learning and managing academic challenges (Ryan & Deci, 2020; Vansteenkiste et al., 2006). To determine the quality of students’ motivation, SDT distinguishes between two forms: autonomous and controlled motivation. When students are driven by autonomous motivation, they experience a sense of volition, and their reasons for studying are perceived as self-directed and self-endorsed. When autonomously motivated, students find the learning material interesting and enjoyable (intrinsic motivation) or personally valuable and meaningful (identified regulation). Intrinsic motivation reflects the most optimal type of motivation, as it is fully autonomous and self-determined (Vansteenkiste et al., 2009). Most studies suggest that higher levels of identified and intrinsic motivation are associated with a number of positive outcomes, such as more positive emotions, better self-concept, and optimal academic achievement (Sutter-Brandenberger et al., 2018; Taylor et al., 2014). In fact, these two forms of motivation are also in positive relation with the use of deep learning strategies (Pekrun, 2013). In contrast, controlled motivation refers to behavior that is driven by pressure to behave in a certain way. With controlled motivation, students experience their study efforts as stemming from internal forms of pressure such as feelings of guilt, shame, or anxiety; the striving for contingent self-approval and pride (introjected regulation); from externally pressuring forces, such as high environmental demands or expectations, the threat of punishments, or the promise of rewards (external regulation; Koole et al., 2019; Ryan & Deci, 2017). In the case of more controlled forms of motivation (external and introjected regulation), the results lack clarity. For example, introjection is related positively to behavioral engagement yet negatively to emotional engagement, and predicts the use of more superficial learning strategies, procrastination, and test anxiety (Boncquet et al., 2024; Van der Kaap-Deeder et al., 2016). We should consider that controlled motivation is not necessarily incompatible with autonomous motivation. It can exist alongside autonomous motivation, as seen in students who exhibit a high level of overall motivation (Vansteenkiste et al., 2009). Controlled motivation presents a somewhat paradoxical impact on students’ learning. While it may yield certain short-term benefits, particularly when it serves as the primary driving force behind university students’ efforts, it ultimately leads to lower-quality learning outcomes compared to autonomous motivation (Boncquet et al., 2024). To sum up, a large number of studies have shown that these distinct forms of academic motivation are related to students’ learning behaviors and academic achievement (Vansteenkiste et al., 2022).

Motivation and academic emotions are also correlated and studied in educational literature (Kim & Pekrun, 2014). While a number of studies have explored the relationship between these constructs (Pekrun, 2013; Sutter-Brandenberger et al., 2018), most literature has traditionally developed along separate lines. Only a few studies have examined academic emotions within the framework of SDT, and these have primarily focused on educational levels other than higher education (Sutter-Brandenberger et al., 2018), or on adults outside the education context (Vandercammen et al., 2013).

### 1.3. Academic Emotions

Academic emotions are defined as affective responses in the context of learning and performance-related activities, typically involving emotions such as anxiety, boredom, and enjoyment (Pekrun et al., 2011). Within the framework of Pekrun’s Control Theory (CVT; Pekrun, 2018), these emotions are considered to play a pivotal role in academic success, as they influence achievement through their impact on motivational, self-regulatory mechanisms, and learning strategies (Pekrun, 2006).

Academic emotions may arise across various educational contexts, including participation in classroom activities (classroom-related emotions), in learning (learning-related emotions), and during assessments (exams-related emotions) (Pekrun, 2006). Three emotions are most frequently experienced in higher education—namely, enjoyment (positive, activating), anxiety (negative, activating), and boredom (negative, deactivating)—during learning. There are two main considerations underlying the selection of these specific emotions. Firstly, these emotions are frequently experienced by university students in achievement settings (Respondek et al., 2017; Pekrun et al., 2002), and secondly, all these three emotions are considered the prime emotions related to academic achievement (Niculescu et al., 2015; Pekrun et al., 2011). Broadly speaking, positive emotions are associated with improved academic achievement, as they foster the use of effective learning strategies and promote greater motivational engagement (Goetz et al., 2012). For example, enjoyment related negatively to high task focus, to effective self-regulation of learning, and to overall deeper learning (Frenzel et al., 2024; Obergriesser & Stoeger, 2020). Conversely, negative emotions are generally linked to diminished motivation and interest, the emergence of task-irrelevant thoughts, and a reduction in the cognitive resources available for effective performance (Daniels et al., 2009; Trigwell et al., 2012). For example, emotions like anger, anxiety, boredom, and shame are related to shallow learning strategies and the less frequent use of metacognitive strategies (Frenzel et al., 2024; Silaj et al., 2021). Nonetheless, these relationships do not always unfold in predictable ways. For example, positive emotions may be linked to the use of surface-level learning strategies, whereas specific negative emotions can facilitate learning processes. (Pekrun et al., 2002; Goetz et al., 2006). Further research is warranted to examine additional factors that may influence the interplay between emotions and learning. In particular, focusing on discrete academic emotions in undergraduate learning contexts, rather than relying on broad categorizations of positive and negative emotions, may offer more nuanced insights into the emotional dynamics of academic engagement.

Enjoyment is conceptualized as a positive activating emotion, falling within the category of activity emotions (Pekrun, 2018). When students perceive a meaningful alignment between their personal goals and the academic tasks, they may experience enjoyment (Linnenbrink, 2007). Furthermore, the experience of enjoyment is typically associated with students’ appraisal of the learning context as meaningful and controllable (Camacho-Morles et al., 2021). It has been shown to be a significant positive predictor of academic performance (Daniels et al., 2009). The academic emotion of anxiety is considered a negative, activating emotion that falls under the category of activating emotion (Pekrun, 2018), and it is frequently reported by university students (Pekrun & Stephens, 2010). Anxiety tends to arise when students assign low value to an achievement situation while perceiving themselves as having moderate control over the outcome. Although anxiety may disrupt focus and hinder performance, it can also motivate increased effort, particularly in emotionally resilient students (Pekrun & Stephens, 2012). Boredom is a negative, deactivating emotion that is categorized as an activity-related emotion (Pekrun, 2018). Students may experience boredom when learning lacks clear goals, when there is a mismatch between personal goals and task demands, or when the activity is perceived as neither negatively nor positively valued (Pekrun et al., 2011). Moreover, perceptions of low control and low value regarding a learning activity have been associated with heightened levels of boredom (Niculescu et al., 2015). It is closely linked to negative effects, diminished interest and stimulation, and a tendency to adopt surface approaches to learning (Pekrun et al., 2010; Sharp et al., 2017).

Pekrun (2018) proposes that students’ academic emotions are primarily shaped by their achievement, control-related appraisals (such as competence beliefs, e.g., self-concept), and value appraisals (beliefs about the intrinsic or extrinsic worth of a subject area, e.g., achievement outcomes). Research has demonstrated that emotions play a crucial role not only in academic achievement, but also in motivation (Sutter-Brandenberger et al., 2018). Emotions also influence individuals’ intrinsic and extrinsic motivation. Positive emotions, especially enjoyment, are crucial catalysts of intrinsic motivation (Isen & Reeve, 2005). On the other hand, negative emotions like anxiety establish a positive correlation with extrinsic motivation or, more broadly, less autonomous forms of motivation. It is important to highlight that studies on emotions and intrinsic/extrinsic motivation from the perspective of self-determination theory have seldom been integrated (for exceptions, see Schwab et al., 2022). University settings generate intense emotions that are directly related to learning processes (Pekrun, 2019).

### 1.4. Student Approaches to Learning

The “student approaches to learning” tradition is one of the main frameworks for comprehending learning in higher education (Entwistle, 2018). Approaches to learning reflect the various ways in which students engage with academic tasks, shaped by their individual characteristics and their perceptions of learning environment (Biggs, 1987). These approaches are typically classified into three distinct types: deep, surface, and organized (Marton & Saljo, 1997; Entwistle et al., 2001). The deep approach, driven by intrinsic motivation, involves active engagement with content and the pursuit of personal understanding, and is positively linked to academic success (Richardson et al., 2012), though this relationship is not consistently observed across all studies (Herrmann et al., 2017). The surface approach is typically driven by extrinsic motivation and involves rote memorization of fragmented content, with minimal cognitive effort. Research has shown that the surface approach to learning is negatively associated with academic achievement (Chamorro-Premuzic et al., 2007; Karagiannopoulou & Milienos, 2013; Karagiannopoulou et al., 2019; Karagiannopoulou et al., 2022). However, in certain demanding learning contexts, the surface approach may serve as an effective coping strategy (Kember, 2004). The third approach, strategic, also known as organized studying, is considered an approach to studying, organizing time and effort and is conceptually similar to self-regulation (Lindblom-Ylänne et al., 2018; Postareff et al., 2017). Some studies have highlighted the positive impact of the strategic approach on academic achievement (Herrmann et al., 2017; Richardson et al., 2012). Research suggests that strategic approaches can complement deep learning, while a lack of organization can affect both deep and surface learning styles (Haarala-Muhonen et al., 2017; Karagiannopoulou & Milienos, 2013). It can be argued that different combinations of approaches to learning are frequently employed among university students (Asikainen et al., 2020).

Until now, few studies have examined approaches to learning along with academic emotions (Rentzios et al., 2019; Karagiannopoulou et al., 2022; Xie & Fan, 2025) and individual factors involved in learning (Karagiannopoulou et al., 2019; Vlachopanou & Karagiannopoulou, 2021). Students experiencing positive emotions during their studies are more likely to adopt a deep approach to learning, whereas those experiencing negative emotions tend to rely on surface approaches (Trigwell et al., 2012). While previous findings align with theoretical expectations, Postareff et al. (2017) emphasize the complex “web” of association among approaches to learning, academic emotions, and study success.

### 1.5. Learning, Emotional, Motivational Factors and University Students’ Profiles

There has been growing research interest in the study of different characteristics in the same groups of individuals. A range of studies show profiles of university students that correspond to adaptive, maladaptive, and dissonant groups (Karagiannopoulou et al., 2020; Milienos et al., 2021; Fryer & Vermunt, 2018). Most studies identify three or four profiles that share specific characteristics. These studies suggest that different factors may coexist that contribute to students’ academic achievement and performance. For example, Parpala et al. (2010) found four discrete profiles based on approaches to learning. The first profile focuses on the strategic approach; they self-regulate their learning based on available time and prioritize their academic tasks. The second profile consists of students who take a deep approach to learning, while the third profile consists mainly of university students who opt for the surface approach to learning. The last profile, which is less distinct, consists of students who do not organize their studies, but exhibit some characteristics of the deep approach. This profile is an example of a “dissonant” group of students whose common variables do not theoretically match (Lindblom-Ylänne & Lonka, 2000). These profiles are frequently identified in the related literature (Parpala et al., 2022); it seems that current research needs to expand the range of factors that should be studied in order to explore this complex phenomenon of “dissonant” profiles. Moreover, Haarala-Muhonen et al. (2017) emphasized that the relationship between approaches to learning and academic achievement is not straightforward. In the same vein, Heikkilä et al. (2011) acknowledge the need to examine a combination of cognitive and emotional factors when researching the quality of learning. In their study, they found three groups of students: the “non-academic” students, the “self-directed” students, and the “helpless” students. The first of the three profiles, the “non-academic”, was the most intriguing. This profile combines several facets that theoretically do not match and are not usually associated with positive academic performance. Nevertheless, students in this group seem to be successful in their academic tasks, they make progress, albeit in a “relaxed” manner and without achieving a high GPA (Heikkilä et al., 2011). This “relaxed” profile was also found in another study where students reported emotions with low valence who did well on their studies (Jarrell et al., 2017). In another relative study, Postareff et al. (2017) found a group of students who expressed negative emotions but still engaged in a deep approach to learning and made significant progress.

This atypical combination of emotional and cognitive factors has provided new insights regarding both learning outcomes and academic performance (Karagiannopoulou & Milienos, 2013; Rentzios, 2021). For example, Karagiannopoulou et al. (2019) identified three profiles based on approaches to learning and defense styles. They found a profile that is named “restricted maturity-dissonant unorganized students” with the lowest GPA, another that is called “defensive-reproduction-oriented students” with mid-low GPA, and lastly, the third that is named “mature and learning-advanced students” with the highest GPA. These profiles correspond to dissonant, surface and deep learners, respectively, with similar scores on adaptive and maladaptive defense styles. Similarly, Vlachopanou and Karagiannopoulou (2021) explored defense styles, academic procrastination, and well-being together with approaches to learning. They found three profiles: (a) “psychologically stable and adaptive” with the greatest GPA, (b) “immature and unstable at risk” with a low GPA, and (c) “defensively dissonant” with a mid-high GPA. The above studies seem to suggest a learning pattern of deep, surface and dissonant learning behaviors. The findings suggest that the stable adaptive deep/strategic group achieves the highest GPA, while surface and low dissonant students report the lowest. Recent studies highlight the complexity of dissonant profiles, where negative affect coexists with otherwise adaptive or successful learning behaviors (Postareff et al., 2017). However, their theoretical interpretation remains limited. Findings from maths anxiety research offer a useful perspective on these apparent contradictions. For example, Cho and Kongo (2024) found that students with high levels of math anxiety can still achieve academic success, though this was frequently associated with reduced interest and a less stable sense of self-efficacy. Similarly, Wang et al. (2018) demonstrated that anxiety does not always impede performance, but may instead contribute to performance via controlled motivation or overcompensation mechanisms. Robinson et al. (2017) likewise identified students who experienced elevated negative affect yet showed strong academic performance in a college science course. Such patterns may help illuminate the functioning of dissonant profiles, where motivation, emotion, learning, and achievement interact in complex ways.

### 1.6. The Present Study

This study aligns with current research indicating that academic emotions, ER, and academic motivation must be considered in research in educational contexts (Pekrun, 2024a). Research clearly suggests that academic settings at universities give rise to intense emotions, and it is feasible to explain them within conceptual frameworks such as CVT, ER theories (Pekrun, 2019) or SDT (Sutter-Brandenberger et al., 2018). Moreover, recent findings suggest that these affective and self-regulatory factors may not only influence students’ academic functioning, but also relate to more consequential outcomes such as dropout ideation, emphasizing their broader impact on academic persistence (Enguídanos et al., 2023).

The integration of these constructs stems from the learning patterns model proposed by Vermunt and Donche (2017), which views learning as the outcome of interrelated processes involving regulation, motivation, emotions, and learning strategies. ER and academic self-regulation depict students’ efforts to regulate their learning, academic emotions function as both drivers and consequences of engagement with learning material, and approaches to learning represent their strategic orientation toward academic tasks. Although these constructs originate from distinct theoretical traditions, bringing them together enables a more comprehensive view of student learning—an approach encouraged by Pekrun (2024b) as necessary for understanding the complexity of learning processes.

Therefore, the aims of the study are twofold: (a) the first aim is to investigate ER, academic emotions, academic motivation, and approaches to learning that individual students experience during their learning process by clustering them on the basis of these variables, and (b) the second aim is to explore differences among clusters with regard to GPA. Based on these, we hypothesize that distinct student profiles will emerge, reflecting combinations of academic emotions, motivational orientations, emotion regulation strategies, and approaches to learning. We expect that profiles characterized by enjoyment, reappraisal, intrinsic motivation, and deep/organized approaches to learning will reflect more adaptive learning patterns and be positively associated with a higher GPA. On the contrary, profiles marked by anxiety, boredom, suppression, external regulation, and a surface approach to learning are expected to be associated with lower GPA. Finally, based on the previous literature, we also anticipate a dissonant profile; a group of students combining theoretically atypical characteristics (e.g., negative academic emotions with deep approaches) (Parpala et al., 2022; Postareff et al., 2017).

## 2. Methodology

### 2.1. Participants and Procedure

A total of 509 undergraduate students from a Greek university participated in the study, who were recruited through convenience sampling. All participants were enrolled in a full-time, four-year degree program, distributed across two academic departments: Social Sciences and Primary Education. The sample comprised 72 male students (13.5%) and 437 female students (86.3%), reflecting the well-documented gender imbalance in these fields within Greek higher education (Eurostat, 2018). The mean age of participants was 20.5 years, with the vast majority (93%) being under 22 years of age. Regarding academic seniority, 136 students (25.8%) were in their first year, 139 (26.4%) in their second, 95 (18%) in their third, and 157 (29.8%) were in their fourth year or beyond. The administration of the questionnaires took place during class time, in coordination with the course instructor and with full respect for the instructional schedule.

### 2.2. Instruments

#### 2.2.1. Demographics

Participants completed a brief demographic questionnaire that gathered information on age, gender, academic department, and year of study.

#### 2.2.2. Academic Emotions

The distinct academic emotions of enjoyment, anxiety, and boredom were assessed using the four learning-related scales of the Achievement Emotions Questionnaire (Pekrun et al., 2011). Participants responded on a five-point Likert scale ranging from 1 (Strongly disagree) to 5 (Strongly agree), evaluating the extent to which they experienced each emotion in relation to studying—either before, during, or after engaging with course material. The enjoyment scale (10 items, e.g., “I look forward to studying”), the anxiety scale (11 items, e.g., “I get tense and nervous while studying”), and the boredom scale (11 items, e.g., “The material bores me to death”). Higher scores on each scale indicate a greater intensity in the experience of that particular emotion. In the present study, internal consistency reliability was acceptable to excellent, with Cronbach’s alpha coefficients of 0.79 for Enjoyment, 0.82 for Anxiety, and 0.92 for Boredom.

#### 2.2.3. Approaches to Learning

Approaches to learning were assessed using the Finnish version of the Approaches to Learning and Studying Inventory (ALSI, Parpala et al., 2013). The instrument comprises 16 items that reflect three distinct approaches to learning: the deep approach (8 items, e.g., “It has been important for me to follow the argument, or to see the reasons behind things.”), the surface approach (4 items, e.g., “I’ve often had trouble making sense of the things I have to remember.”), and the organized approach (4 items, e.g., “On the whole, I’ve been quite systematic and organized in my studying”). Participants responded to each item using a five-point Likert scale (1 = Strongly disagree to 5 = Strongly agree). Higher scores on each subscale indicate a stronger preference for each approach. The inventory has been translated into Greek and has demonstrated satisfactory psychometric properties (Karagiannopoulou et al., 2014; Rentzios et al., 2019). In the current study, Cronbach’s alpha coefficients were 0.74 for the deep approach, 0.75 for the surface approach, and 0.82 for the organized approach, indicating acceptable to good internal consistency.

#### 2.2.4. Emotion Regulation

The Emotion Regulation Questionnaire (ERQ) (Gross & John, 2003) was used to measure cognitive ER. It is a 10-item measure that assesses two common emotion regulation strategies: cognitive reappraisal (6 items) and expressive suppression (4 items). Responses are on a 7-point Likert scale, ranging from “strongly disagree” (=1) to “strongly agree” (=7). Example items for cognitive reappraisal include statements like “When I want to feel more positive emotion (such as joy or amusement), I change what I’m thinking about,” while for expressive suppression, an example is “I keep my emotions to myself.”

#### 2.2.5. Academic Self-Regulation/Motivation

The motivations of students for studying were evaluated using a modified version of the Academic Self-Regulation Scale (Ryan & Connell, 1989). The 16-item scale, which contains 4 items per regulation, asks participants to state their reason for studying. The measure consists of four subscales, tapping four different types of motivation for studying—that is, external regulation (e.g., “Because I’m supposed to do so”; 4 items); introjected regulation (e.g., “Because I want others to think I’m smart”; 4 items); identified regulation (e.g., “Because I want to learn new things”; 4 items); and intrinsic motivation (e.g., “Because I enjoy doing it”; 4 items). Participants were asked to indicate their agreement with the items on a 5-point Likert scale ranging from 1 (totally disagree) to 5 (totally agree).

### 2.3. Statistical Analysis

The latent structure of the variables of interest was assessed by Confirmatory Factor Analysis (CFA). Pearson’s correlation coefficient was used to measure the correlation among the variables. Moreover, Cronbach’s α was calculated in order to evaluate the reliability of the data.

A hierarchical cluster analysis was performed to identify groups of individuals with similar profiles across the selected variables. Prior to clustering, all variables were standardized using z-score normalization to ensure comparability across scales. Pairwise distances between observations were then calculated using the Euclidean distance metric.

Several agglomerative methods were tested, including average, single, complete, and Ward’s linkage. Based on the agglomerative coefficient, Ward’s method was selected, as it provided the most coherent clustering structure.

The optimal number of clusters was determined using two complementary approaches: the gap statistic and a multi-index approach based on a comprehensive evaluation of established clustering indices, including the Hubert index and the D index (Hubert & Arabie, 1985; Tibshirani et al., 2001). Following the majority rule across these indices, we identified the most appropriate number of clusters.

Analysis of Variance (ANOVA) was used to examine differences of the variables of interest among clusters, as well as the difference of GPA of the participants among clusters. Post-hoc multiple tests, specifically Tukey’s HSD, examined the pairwise comparisons in case of statistically significant results from ANOVA, adjusting for multiple comparisons. Furthermore, ANOVA was also used to explore the age differences between the clusters, and Pearson’s chi-square was used to examine the sex differences among clusters. Discriminant and decision tree analyses were employed as validation techniques to assess the clarity and predictive separability of the derived clusters. Discriminant Analysis was used both to support the statistical robustness of the cluster solution and to provide a deeper understanding of the findings from ANOVA. Finally, a Decision Tree model using the Exhaustive CHAID as growing method and with GPA as the dependent variable was employed to assess whether cluster membership outperformed the individual psychological variables in predicting GPA, thereby validating the explanatory power of the derived profiles. All statistical analyses were carried out using IBM SPSS v.29 and the R programming language (Version 4.4.3).

## 3. Results

Descriptive statistics for the academic emotion variables (Enjoyment, Anxiety and Boredom), the ER variables (Reappraisal and Suppression), the academic self-regulation variables (External, Introjected, Identified and Intrinsic), and the approaches to learning variables (Deep, Surface and Organized) can be found in Table 1. CFA results for the aforementioned variables are presented in Table 2. Overall, the majority of the indices fall within acceptable value ranges, confirming the validity of the instruments’ latent structures. More particularly, CFI, GFI, NFI, and TLI exhibit high values (>0.90), whereas RMSEA and SRMR demonstrate low values (most of them <0.05).

The results of Pearson’s correlation coefficient and Cronbach’s α are presented in Table 3. Overall, Cronbach’s α was greater than 0.74 for all variables of interest. Regarding the correlations among the variables of interest, enjoyment was highly positively correlated with the identified, intrinsic, and deep variables, and highly negatively correlated with boredom. Anxiety was highly positive correlated with the boredom and surface variables. External regulation was highly positively correlated with the introjected variable, and the identified variable was highly positively correlated with the intrinsic variable.

**Figure 1:**
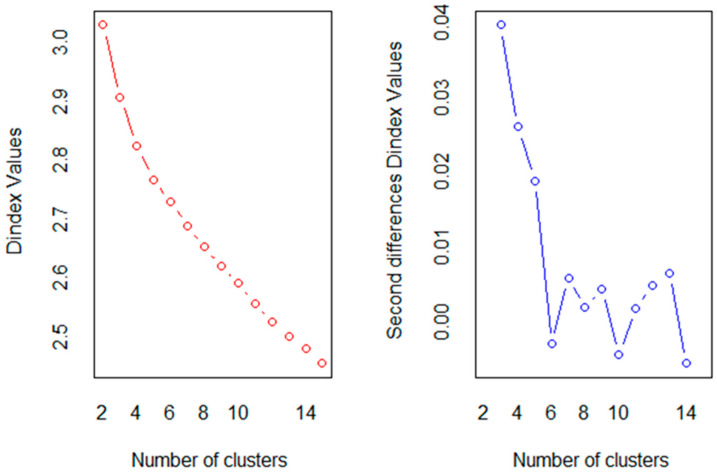
Optimal number of clusters based on Hubert and D indexes.

Using Agglomerative Nesting Hierarchical Clustering, we classified the participants into homogeneous groups based on their scores on variables associated with academic emotions, ER, academic self-regulation, and approaches to learning. Clustering was based on the dissimilarity matrix computed by the Euclidean distance, and the Ward method was performed. The Hubert and the D indexes, which are used for determining the optimal number of clusters, suggested that the three-cluster is the best solution (Figure 1).

**Figure 2:**
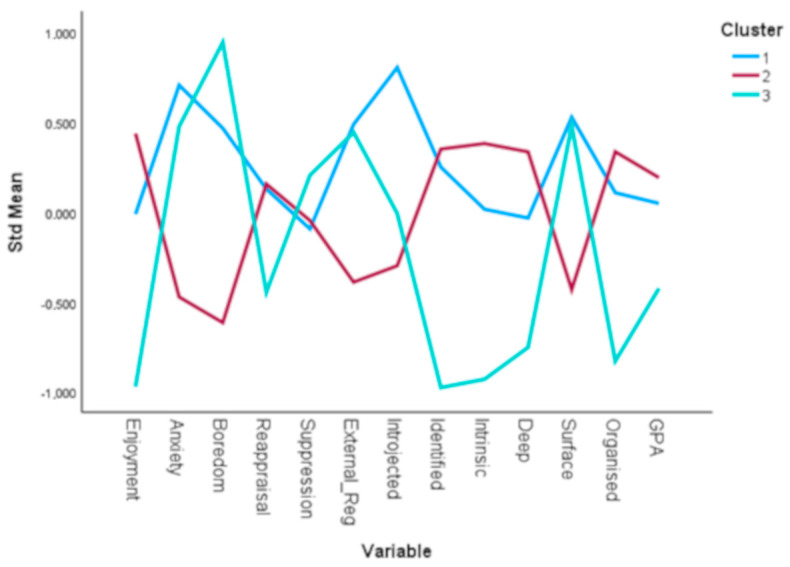
Standardized mean scores of study variables across the three clusters.

Regarding demographic differences between clusters, ANOVA revealed statistically significant differences in participants’ age (*p*-value < 0.05), with post-hoc comparisons indicating that students in cluster 2 were slightly older than those in cluster 1. Additionally, a significant difference was observed in gender distribution across the three clusters (*p*-value = 0.008), with a higher proportion of male students found in clusters 2 and 3, while female students were more evenly distributed across all clusters. Table 4 presents the mean values of the study variables across the three clusters. ANOVA followed by post-hoc comparisons revealed statistically significant differences between clusters for all variables (*p*-value < 0.05). In more detail, participants in cluster 2 had significantly higher mean scores in the enjoyment, intrinsic, and deep variables, and significantly lower scores in the anxiety, boredom, external regulation, introjected, and surface variables. Cluster 3 was characterized by significantly higher mean scores in boredom and significantly lower scores in the enjoyment, reappraisal, identified, intrinsic, deep and organized variables. Finally, cluster 1 contains participants with significantly higher scores in the introjected variable. ANOVA was also used to examine statistically significant differences in GPA across the three clusters. The results indicated a significant difference across the three clusters (*p*-value < 0.05). These differences are also visually depicted in Figure 2, which presents a line plot of standardized mean scores across the three clusters.

Regarding the age of the participants, ANOVA showed statistically significant differentiations of the mean age among the clusters (*p*-value < 0.05). Post-hoc comparisons demonstrated that there is a statistically significant difference in mean age among Cluster 1 and Cluster 2 (19.51 years vs. 19.97 years; *p*-value = 0.024; Table 5). Moreover, Pearson’s Chi-square test revealed that sex was not equally distributed among the clusters (*p*-value = 0.008). Male participants were distributed as 16.2%, 42.6% and 41.2% among the three clusters, respectively, and female participants were distributed as 24%, 52.4% and 23.6% among the three clusters, respectively (Table 4). More specifically, 83.8% of the males are placed in clusters 2 and 3, and regarding female participants, 52.4% are placed in cluster 2 and the rest, 47.6%, in clusters 1 and 3.

Discriminant analysis was performed to provide a more detailed insight into the ANOVA findings. The two discriminant functions were statistically significant, meaning that they both contribute to participant classification and should be interpreted (*p*-values < 0.01; Table 5). The first function explains 76.8% while the second explains 23.2% of the variance. The first function is mainly affected by the boredom, organized (negatively), deep (negatively), external regulation, and anxiety variables, whereas the second function is mainly affected by the introjected and identified regulation variables. Additionally, based on the classification results, 88% of cases are correctly classified to their clusters. Moreover, in Table 5, we can observe that cluster 1 consists of students who have a positive mean at both functions (0.667, 1.263). This means that students in cluster 1 have large values in anxiety, boredom, introjected, identified. Cluster 2 contains those who have a negative mean at both functions (−1.22, −0.216). This means that students in cluster 2 have large values in enjoyment, intrinsic, deep and organized. Finally, cluster 3 consists of students who have a positive mean at the first function and a negative at the second function (1.817, −0.701). This means that students in cluster 3 have low values in the enjoyment, reappraisal, identified, intrinsic, deep, and organized variables.

**Figure 3:**
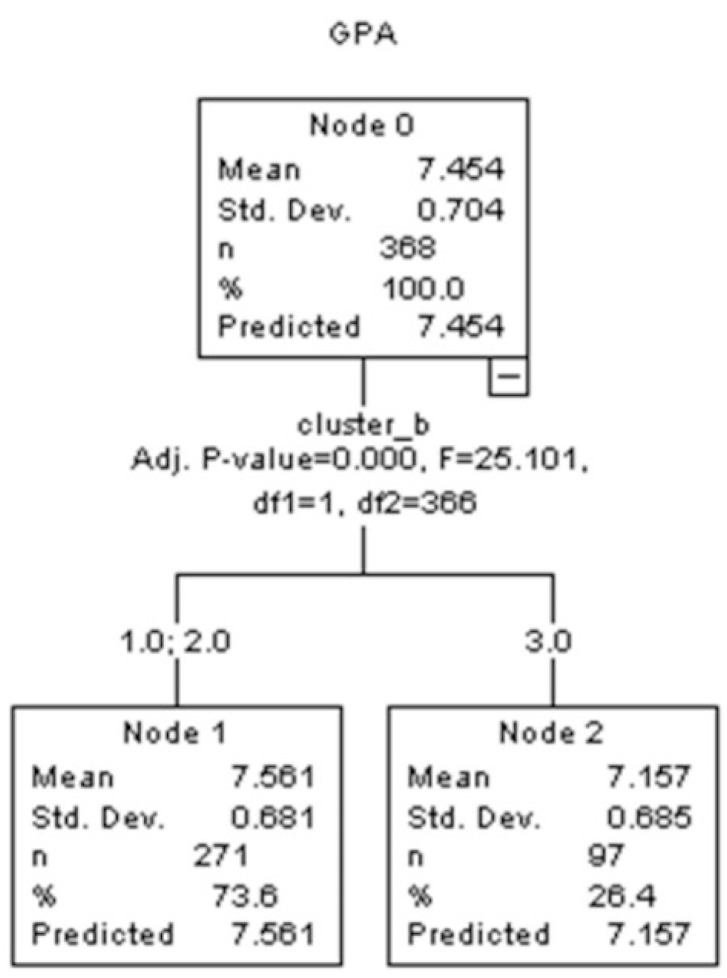
Decision tree with “exhaustive CHAID” being the growing method.

Finally, a decision tree model with the GPA as the dependent variable, examined the contribution of the variables of interest and the cluster membership to the GPA. The results of the decision tree indicated that only the cluster membership influenced GPA, highlighting the strong predictive value of the clustering solution (Figure 3). In more detail, participants in cluster 3 (n = 97) have a mean GPA of 7.157, while participants of the first two clusters (n = 271) have a higher mean GPA of 7.561.

## 4. Discussion

This study examines the emotional-learning profiles of university students, integrating approaches to learning with academic emotions, emotion regulation strategies, and academic motivation. It draws on recent perspectives about the combination of ER and academic emotions (Harley et al., 2019), while also involving SDT (Ryan & Deci, 2020) and approaches to learning. The findings are aligned with current research indicating that, to obtain a more holistic understanding of university students’ learning and academic performance, it is essential to concurrently examine academic emotions, ER, and motivation within educational contexts (Pekrun, 2024a). The study suggests three distinct profiles: the “Anxious, effectively-engaged, and organized learners” (Cluster 1), the “Deep, Happy, and intrinsically motivated learners” (Cluster 2), and the “The Disengaged, Bored, and Suppressing Learners” (Cluster 3). This three-profile solution, which focuses on a combination of emotional, motivational, and learning factors, corroborates previous studies that highlight the importance of emotions and ER variables in the learning process (Karagiannopoulou et al., 2020; Rentzios & Karagiannopoulou, 2021).

### 4.1. “Anxious, Effectively-Engaged, and Organized Learners: Cluster 1”

The first cluster (Cluster 1), the “Anxious, effectively-engaged, and organized learners”, provides new insights into the relationship between the academic emotion of anxiety, academic motivation, and learning behavior. This group is characterized by high scores in anxiety, which unsurprisingly comes along with mid-level scores in a deep approach and high scores in surface and organized approaches to learning. Interestingly, these approaches to learning co-occur with high scores in both extrinsic and identified regulation alongside intermediate introjected regulation. This profile highlights the complexity of learning in the presence of demanding academic settings (Postareff et al., 2017) and reveals a mixture of both motivation and learning approaches. It depicts a “dissonant” group of university students; the variables comprising this cluster do not theoretically fit together (Lindblom-Ylänne & Lonka, 2000; Parpala et al., 2022). Similar patterns have also been observed in studies exploring math anxiety and science courses, where students with high anxiety demonstrate strong performance, often relying on externally regulated motivation and strategic coping mechanisms (Cho & Kongo, 2024; Robinson et al., 2017). This suggests that the interplay of controlled motivation and anxiety may underline such dissonant profiles. A similar pattern was observed by Wünsch et al. (2025), who showed that students under stress may still perform well when they perceive a task as controllable, regardless of its actual difficulty.

Possibly, the mid-level score in a deep approach to learning demonstrates an inherent interest in understanding new material and assimilating knowledge deeply (Hailikari et al., 2022). However, this comes along with the highest scores in surface and organized approaches. Such a dissonant combination appears to co-exist with high scores in both external and identified motivation along with mid-level scores in introjected motivation. Students seem to use a reproduction orientation (Vermunt & Donche, 2017) followed by some degree of personal understanding while they appear to have reached identified motivation, which implies a degree of personal engagement with learning and understanding the rationale of studying as their own. This combination of approaches to learning is associated with a high GPA (similarly high to that reported by students in cluster 2), and in the experience of a degree of enjoyment about their learning. However, the co-occurrence of low intrinsic motivation and a degree of introjected motivation may be related to academic anxiety, which appears to be corroborated in the use of a surface approach. ANOVA and discriminant analysis consistently highlighted this pattern, indicating particularly high scores for anxiety, as well as introjected and identified motivation in this group of students. This result is further supported by their poor use of emotion regulation strategies; they scored the lowest in suppression and reported a mid-level score in reappraisal. This pattern may be interpreted as reflecting less-developed ER skills (Martin & Ochsner, 2016); this group reported the lower average age.

In conclusion, the “Anxiously, effectively-engaged, and organized learners”, comprising a dissonant profile, strive to maintain a high GPA, utilizing all approaches to learning and sustaining an introjected motivation that is likely to support this goal. However, the second score in academic enjoyment, combined with the highest score in anxiety during learning, suggests an emotional burden that comes at a cost in their effort to achieve good grades, or even to acquire the ability to deeply engage and understand the learning material. It could be suggested that the need and the demanding motive to succeed may yield immediate results, but also may undermine long-term well-being and the ability to self-regulate their learning (Vansteenkiste et al., 2018).

### 4.2. “Deep, Happy, and Intrinsically Motivated Learners”: Cluster 2

The second profile of our study, “the Deep, Happy, and intrinsically motivated learners,” were marked by the highest levels of academic emotion in enjoyment, intrinsic motivation for learning, deep approach to learning, and organized studying. Not surprisingly, students in this cluster scored the lowest in the surface approach and also in anxiety and boredom during studying. This profile represents an emotionally adaptive and highly self-regulated group of deep learners who engage effectively with academic demands, reflecting an emotionally and cognitively well-balanced deep profile. This profile is consistent with previous studies that explore similar variables (Karagiannopoulou et al., 2020; Milienos et al., 2021; Postareff et al., 2017).

The combination of deep and organized approaches is the most typical combination in every discipline at the university (Parpala et al., 2022); university students typically achieve the highest scores on the deep approach and the lowest on the surface approach (Herrmann et al., 2017). In our study, this group is the largest. Furthermore, the highest level of intrinsic motivation in this group signifies that these students are motivated by a genuine interest followed by enjoyment for the engagement with the learning material depicted in deep learning. Intrinsic motivation drives a deep learning approach (Kyndt et al., 2010) while the positive activating emotion of enjoyment is linked to greater persistence and effort, which may support deeper engagement with the learning process that is possibly reflected in high scores in organized study. Enjoyment remains a pivotal affective component associated with students’ motivation and engagement while keeping the level of focus on high standards in academic settings (Pekrun & Perry, 2014). This, when combined with an organized study (Entwistle, 2018), results in a higher GPA. ANOVA and discriminant analysis confirmed that these individuals demonstrated large values in enjoyment, deep, and organized study. In our study, this group of students who scored high in both deep and organized approaches reported the highest GPA, a result that is corroborated in other studies too (Karagiannopoulou et al., 2019; Ning & Downing, 2015).

Students in this cluster seem emotionally adaptive and effective in ER. They scored the highest in enjoyment and the lowest in anxiety and boredom. Moreover, they scored the highest in reappraisal and the lowest in suppression. They seem to adequately regulate their negative emotions while they keep enjoyment during learning. Effective ER during learning helps university students remain calm and focused on their tasks (Harley et al., 2019; Webster & Hadwin, 2015). Possibly, they pursue learning for its own sake, finding personal meaning in academic tasks rather than being influenced by external demands or internalized pressures. This intrinsic motivation aligns with high enjoyment and low boredom, as these individuals experience mostly positive emotions that keep their curiosity and commitment in the learning material. From this perspective, adaptive ER and dominance of positive emotions in comparison to negative ones (anxiety and boredom) allow students to analyze and interpret knowledge through comprehending the bigger picture (Parpala et al., 2022), free from the inhibition imposed by negative emotions. We suggest that an adaptive emotional state may support learning and achievement; it allows the employment of deep learning, along with the use of effective time management skills and goal-setting strategies to maximize the learning process, leading to a higher GPA. Such an interpretation sheds light on marginal differences in GPA with students in cluster 2 reporting the highest.

Overall, the “Deep, Happy, and intrinsically motivated learners” show a typical mix of the best characteristics for academic emotions, ER, academic motivation, and learning styles. This is a profile that illustrates how the co-occurrence of adaptive emotional, motivational, and cognitive facets may relate to academic and emotional well-being.

### 4.3. “The Disengaged, Bored, and Suppressing Learners”: Cluster 3

This profile is marked by the highest scores in boredom and suppression and the lowest score in enjoyment, reappraisal, identified and intrinsic motivation, and deep and organized approach. It is a cluster that comprises the negative qualities of emotional, motivational, and learning constructs. Not surprisingly, these students reported the lowest GPA. We suggest that this group of students is at risk both in terms of learning and emotional experiences (Milienos et al., 2021).

In the context of interplay between learning and emotions, these students scored high in the surface approach to learning while scoring the lowest in deep and organized approaches. Such a reproducing approach came along with distinctively high boredom and high anxiety; anxiety in learning has been found to go hand in hand with the surface approach (Rentzios & Karagiannopoulou, 2021; Trigwell et al., 2012). Recent studies associate negative academic emotions with suppression (Karagiannopoulou et al., 2022). In our study, students in this group scored the highest in suppression, while they scored the lowest in reappraisal. Although in some cases, suppression may act as a beneficial factor helping individuals control their emotions and be essential in the academic context (Burić et al., 2016), in our study the use of suppression comes along with lower levels of both deep and organized approaches to learning. A similar pattern was found in the study from Ben-Eliyahu and Linnenbrink-Garcia (2015); they reported that suppressing emotions may hinder the effectiveness of learning strategies, as it demands greater regulatory resources. Possibly, in order to regulate negative emotions, students deploy suppression at the cost of a deep approach which demands an interrogative stance in learning as a prerequisite for curiosity and exploration (Desatnik et al., 2023; Karagiannopoulou et al., in press). Moreover, this interplay between learning and emotion is further supported by the lowest GPA, the dominance of boredom along with the lowest scores in enjoyment, in identified motivation, and in intrinsic motivation. Boredom, a “silent emotion” in comparison to other emotions, in academic environments has been widely associated with low intrinsic motivation and lack of interest (Pekrun et al., 2010). Moreover, it affects a wide range of constructs such as cognition, motivation, learning strategies, and academic performance (Pekrun, 2018; Tze et al., 2013). High levels of boredom may be linked to lower engagement in organized studying, which is considered a critical factor in supporting university students’ well-being and academic achievement (Asikainen et al., 2014).

Their limited experience of enjoyment compared to other groups may further undermine their engagement with the learning material. This emotional detachment possibly depicts disengagement from the learning process; they scored the lowest in organized approach and intrinsic motivation. Research has shown that unorganized students could experience more study-related burnout (Asikainen et al., 2020). Besides, intrinsic motivation encourages individuals to engage in learning willingly, with interest, enthusiasm, and curiosity, considering this the most desirable form of motivation (Vansteenkiste et al., 2018). Students in this group scored the lowest in intrinsic motivation, suggesting that they lack a sense of volitional motivation toward the pursuit of their academic goals. The high scores on both external and introjected regulations demonstrate that their motivation stems from internalized demands and external pressure, a combination that usually undermines long-term objectives (Ryan & Deci, 2020). With the absence of intrinsic motivation, these university students perceive the academic activities as meaningless; possibly experiencing a strong emotional burden that co-exists with heightened boredom and disengagement from learning as reflected in the low GPA. ANOVA and discriminant analysis revealed that participants in this group have low values in enjoyment, reappraisal, intrinsic, deep, and organized study.

In conclusion, the “The Disengaged, Bored, and Suppressing Learners” represents a combination of maladaptive learning and motivational characteristics along with the dominant presence of boredom and suppression. These students approach learning with the minimum enjoyment and enthusiasm, with no organized techniques or the need to meaningfully understand learning material; they rely mainly on the surface approach to learning and struggle to manage their emotions through the ER of suppression. We suggest that this group of students, due to lack of interest and a combination of anxiety and high boredom, can be a target group for intervention to ensure their support.

### 4.4. Limitations and Future Research

Although the study provides novel insights into the complex relations among academic emotions, ER, motivation, and approaches to learning. Nevertheless, several limitations must be acknowledged. Firstly, the self-report methodology, although widely recognized, fails to provide a more nuanced picture of the interaction between the constructs examined in this study. Experimental or longitudinal studies may yield further information. Another restriction is the unbalanced representation of female participants in the study, resulting in a considerable gender imbalance favoring women; unfortunately, this imbalance is the norm in social science departments from which the participants were drawn (Eurostat, 2018). Future research could replicate the present study in academic departments with a higher proportion of male students, to explore whether similar learning profiles emerge in predominantly male populations. Future research may further explore dissonance, frequently noted in educational literature (Karagiannopoulou et al., 2019, 2020; Parpala et al., 2022; Rentzios, 2021), by analyzing additional individual factors that appear more distal and are not seen to align theoretically, such as attachment, mentalizing, and epistemic trust, and their relationship to learning. Lastly, future studies may also benefit from a more systematic approach to variable selection; using theory-based strategies to identify key variables could improve the clarity of student profile interpretations.

## 5. Conclusions

Our findings highlight the importance of creating supportive learning environments that focus on academic emotions and their regulation, and fostering intrinsic motivation. Moreover, our study sheds light on the value of a person-centered approach to comprehending the complex interplay among emotions, ER, motivation, and learning. Each profile highlights distinct emotional and motivational dynamics, emphasizing that seeing the “one way approach” to support and teaching is not enough. Pekrun (2024b) argues that in order to capture the complexity of learning processes, meaningful integration of constructs across theoretical traditions is both necessary and possible. The way students deal with their emerged academic emotions matters not only for their academic achievement, but also for their psychical health and well-being; the importance of emotions and ER in learning settings is critical and thus should be addressed in earlier educational stages (Stockinger et al., 2025). Through psychoeducational interventions, universities should foster intrinsic motivation and enhance emotion regulation strategies. Such strategies have also been shown to buffer the impact of negative emotions on critical outcomes like dropout ideation, underlining their practical value in student support (Enguídanos et al., 2023). These measures will help students, tutors, and academic advisors understand the complex interplay of emotions and learning behaviors that emerge under demanding academic settings.

## Figures and Tables

**Table 1:** Descriptive statistics of variables of interest.

	N	Mean	Std. Dev.	Min	Max
Enjoyment	509	32.845	5.084	9.1	45.5
Anxiety	508	28.597	7.265	10.091	50.455
Boredom	509	25.49	8.861	10.091	50.455
Reappraisal *	509	−0.005	0.995	−3.611	1.834
Suppression *	509	0.009	0.999	−1.839	2.873
External Reg	509	2.248	0.804	1	5
Introjected	509	2.499	0.908	1	5
Identified	509	4.452	0.662	1	8
Intrinsic	509	3.442	0.867	1	6
Deep	508	3.855	0.535	1.75	5
Surface	508	2.882	0.835	1	5
Organized	508	3.605	0.871	1	5
GPA	368	7.454	0.704	5.4	9.5

* Reappraisal, suppression are standardized values.

**Table 2:** Confirmatory factor analysis on the instruments utilized.

	CFI	GFI	AGFI	NFI	TLI	RMSE (90% CI)	SRMR
Enjoyment	0.946				0.907	0.070 (0.054, 0.086)	0.048
Anxiety	0.952				0.930	0.054 (0.041, 0.068)	0.038
Boredom	0.968				0.953	0.073 (0.060, 0.086)	0.028
Emotion Regulation	0.972	0.973		0.952	0.961	0.050 (0.035, 0.065)	0.036
Academic Self-Regulation	0.967			0.949	0.958	0.056 (0.048, 0.065)	0.049
Approaches to Learning	0.956	0.956			0.945	0.044 (0.035, 0.053)	0.047

**Table 3:** Cronbach’s alpha (α) and Pearson correlation coefficient among the variables of interest.

	α	Enjoyment	Anxiety	Boredom	Reappraisal	Suppression	External_Reg	Introjected	Identified	Intrinsic	Deep	Surface	Organized
Enjoyment	0.79	1	−0.310 **	−0.562 **	0.245 **	−0.118 **	−0.275 **	−0.043	0.600 **	0.623 **	0.575 **	−0.336 **	0.356 **
Anxiety	0.82		1	0.563 **	−0.075	0.072	0.317 **	0.371 **	−0.220 **	−0.308 **	−0.163 **	0.517 **	−0.132 **
**Boredom_**	0.92			1	−0.147 **	0.050	0.327 **	0.181 **	−0.465 **	−0.492 **	−0.372 **	0.490 **	−0.400 **
**Reappraisal**	0.80				1	0.000	−0.113 **	0.034	0.211 **	0.236 **	0.303 **	−0.107 *	0.135 **
**Suppression**	0.74					1	0.174 **	0.145 **	−0.103 *	−0.037	−0.007	0.057	−0.076
**External_Reg**	0.79						1	0.512 **	−0.337 **	−0.318 **	−0.204 **	0.251 **	−0.148 **
**Introjected**	0.76							1	−0.091 *	−0.147 **	−0.018	0.185 **	−0.022
**Identified**	0.87								1	0.640 **	0.408 **	−0.216 **	0.280 **
**Intrinsic**	0.88									1	0.449 **	−0.306 **	0.294 **
**Deep**	0.74										1	−0.194 **	0.299 **
**Surface**	0.75											1	−0.128 **
**Organized**	0.82												1

*: *p*-value < 0.05; **: *p*-value < 0.01.

**Table 4:** Descriptive statistics for the variables of interest at each cluster.

	Clusters				
	1 (n = 117)	2 (n = 260)	3 (n = 132)	*p*-Value	Partial η^2^
Enjoyment, mean	32.946	35.253	28.013	<0.001 ^1^	0.349
Anxiety, mean	33.508	24.769	31.807	<0.001 ^1^	0.298
Boredom, mean	29.34	19.628	33.626	<0.001 ^1^	0.486
Reappraisal, mean ^3^	0.129	0.157	−0.444	<0.001 ^1^	0.068
Suppression, mean ^3^	−0.092	−0.046	0.206	<0.028 ^1^	0.013
External_Reg, mean	2.62	1.911	2.583	<0.001 ^1^	0.184
Introjected, mean	3.209	2.197	2.464	<0.001 ^1^	0.198
Identified, mean	4.628	4.694	3.82	<0.001 ^1^	0.321
Intrinsic, mean	3.494	3.815	2.661	<0.001 ^1^	0.306
Deep, mean	3.855	4.054	3.465	<0.001 ^1^	0.208
Surface, mean	3.301	2.5	3.261	<0.001 ^1^	0.218
Organized, mean	3.716	3.915	2.898	<0.001 ^1^	0.240
GPA, mean	7.489	7.59	7.157	<0.001 ^1^	0.067
Age, mean	19.513	19.969	19.848	<0.032 ^1^	0.013
Gender					
Females, n (%)	106 (24)	231 (52.4)	104 (23.6)	<0.008 ^2^	
Males, n (%)	11 (16.2)	29 (42.6)	28 (41.2)		

^1^ ANOVA ^2^ Chi-square (χ^2^) ^3^ Reappraisal, suppression are standardized values.

**Table 5:** Discriminant analysis results.

	Stand. Canonical Discriminant Function Coefficients		Structure Matrix	
	Function		Function	
	1	2	1	2
Enjoyment	−0.130	0.039	−0.534	0.300
Anxiety	0.200	0.214	0.445	0.403
Boredom_	0.492	0.226	0.744	0.084
Reappraisal	−0.099	0.139	−0.174	0.204
Suppression	0.011	−0.216	0.067	−0.112
External_Reg	0.205	0.131	0.340	0.224
Introjected	0.131	0.593	0.203	0.585
Identified	−0.070	0.551	−0.448	0.492
Intrinsic	−0.158	0.178	−0.478	0.309
Deep	−0.211	−0.062	−0.379	0.200
Surface	0.152	0.163	0.380	0.247
Organized	−0.301	0.315	−0.388	0.317
Eigenvalue	1.727	0.522		
% Variance	76.8	23.2		
	Functions at group centroids			
Clusters	1	2		
1	0.667	1.263		
2	−1.220	−0.216		
3	1.817	−0.701		
	Wilks’ Lambda			
Test of functions	Wilks’ Lambda	Chi-square	df	*p*-value
1 trough 2	0.241	709.505	24	<0.001
2	0.657	209.490	11	<0.001

## Data Availability

The datasets used and/or analyzed during the current study are available from the corresponding author on reasonable request.
